# How I Teach It: The Ross Procedure Using Explanted Hearts During Orthotopic Heart Transplantation

**DOI:** 10.1016/j.cjco.2023.07.007

**Published:** 2023-07-20

**Authors:** Raghav Chandra, Christopher A. Heid, Ishwar Chuckaree, Nicholas Marshall, Michael A. Wait, Matthias Peltz, John S. Murala

**Affiliations:** a Department of Surgery, University of Texas Southwestern Medical Center, Dallas, Texas, USA; b Department of Cardiovascular and Thoracic Surgery, University of Texas Southwestern Medical Center, Dallas, Texas, USA; c School of Medicine, University of Texas Southwestern Medical Center, Dallas, Texas, USA

**The Ross Procedure (RP) is a technically complex operation for aortic valve replacement (AVR) in selected patients. We describe a novel approach to simulating this operation using explanted native hearts during heart transplantation. Consent was obtained. The aortic root is dissected away from the pulmonary artery (PA). The pulmonary autograft is harvested and then** **transferred** **into the aortic position, and coronary buttons are reimplanted. The explanted heart is submitted for pathologic analysis. Six explanted hearts have been used for simulation thus far. Participating trainees reported high degrees of satisfaction. This high-fidelity simulation is an effective way to increase trainee exposure to the RP.**

The RP is a complex operation for younger patients with aortic valvular disease in which the aortic valve and root are replaced with the patient’s pulmonic valve, and the pulmonic valve is replaced with a homograft.^1^ This approach obviates the need for lifelong anticoagulation as would be required for mechanical valve replacement, and it avoids the structural valve degeneration seen in bioprosthetic valves.^2^ Large meta-analyses have demonstrated that the use of the RP in children and young adults is associated with 90%-95% of the life expectancy of that of an age- and sex-matched general population.^3^ Recent investigations demonstrate that, compared to traditional mechanical AVR, the RP is associated with reduced risk of postoperative stroke and major bleeding, as well as reduced risk of endocarditis and reduced need for reoperation compared to bioprosthetic AVR.^2^ Furthermore, the overall mortality risk may be lower with the RP.^2^ A major feature of the RP is that the autografted pulmonic valve is able to adapt to somatic growth of the aortic root with age, which preserves its structural integrity.^3^ Pulmonic autograft implantation may be associated with more physiologic flow dynamics through the aortic root and lower mean gradients. This approach may lead to reduced risk of heart failure compared to traditional AVR, in which the annulus is static.^4^ An important point to note is that RP is associated with high rates of reintervention with respect to both long-term dilatation of the autograft, which may outstrip somatic growth, and right ventricular outflow tract (RVOT) conduit degeneration.^3^ A point worth noting is that the RP is associated with re-establishing an age-matched life expectancy in young people with aortic valve and/or root pathology.^2^ Although the primary pathophysiologic process driving autograft deterioration is dilatation of the neo-aortic root, the technical complexity of the RP also may be implicated significantly in the variability of autograft durability.^3^

Given the above collective advantages, the RP is recommended for young patients requiring aortic valve replacement.^2^ However, the RP is technically complex and is performed infrequently, often by single surgeons at select high-volume institutions. As the population of younger patients with aortic valvular disease lives longer, the next generation of cardiothoracic surgeons need to be trained in performing the RP. To improve trainee exposure to this operation, our institution has adopted a novel approach to teaching the RP using freshly explanted adult hearts at orthotopic heart transplantation (OHT).

## Materials and Methods

**Figure 1:**
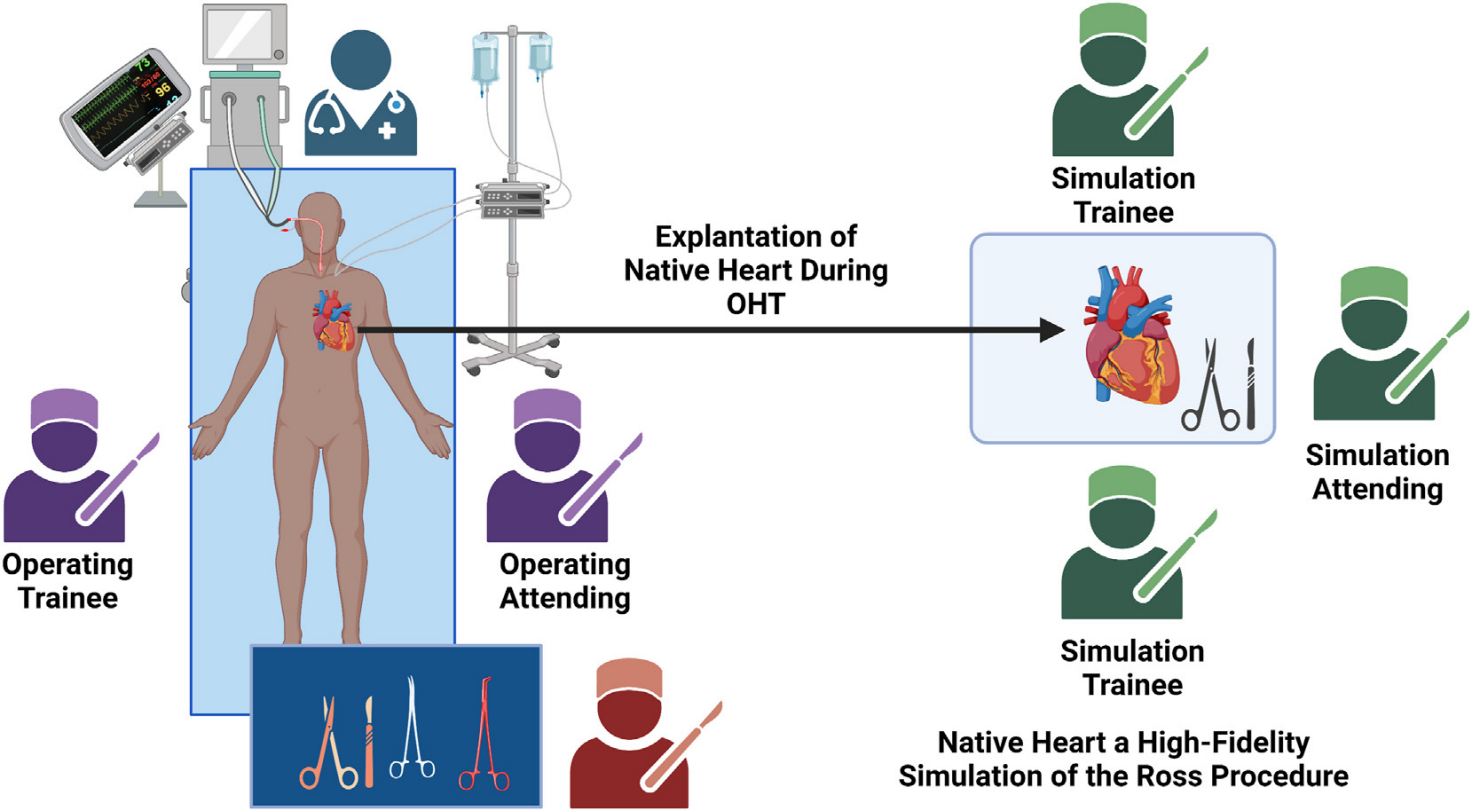
Setup for the Ross Procedure simulation during orthotopic heart transplantation (OHT). While the operating team conducts the transplantation, the simulation attending, most frequently the procurement surgeon, leads resident and fellow trainees through the Ross procedure simulation, using the native explanted heart as a high-fidelity model. Created with BioRender (BioRender.com).

Traditionally, following recipient cardiectomy, the explanted heart is sent to the pathology laboratory for final evaluation. To optimize resident and fellow education, we utilize these specimens in the operating room immediately following explant, to teach the RP. Any trainees who do not participate actively in the OHT can learn this complex operation using the recipient heart. This simulation is facilitated by a second attending surgeon who has dedicated training in congenital heart disease and the RP (Fig. 1). The explanted heart serves as a high-fidelity model for learning, with no risk to the patient. The final specimen still is sent for pathologic analysis in standard fashion. Here, we describe our approach to teaching the operation. Institutional review board approval and informed consent from patients were obtained.

**Figure 2:**
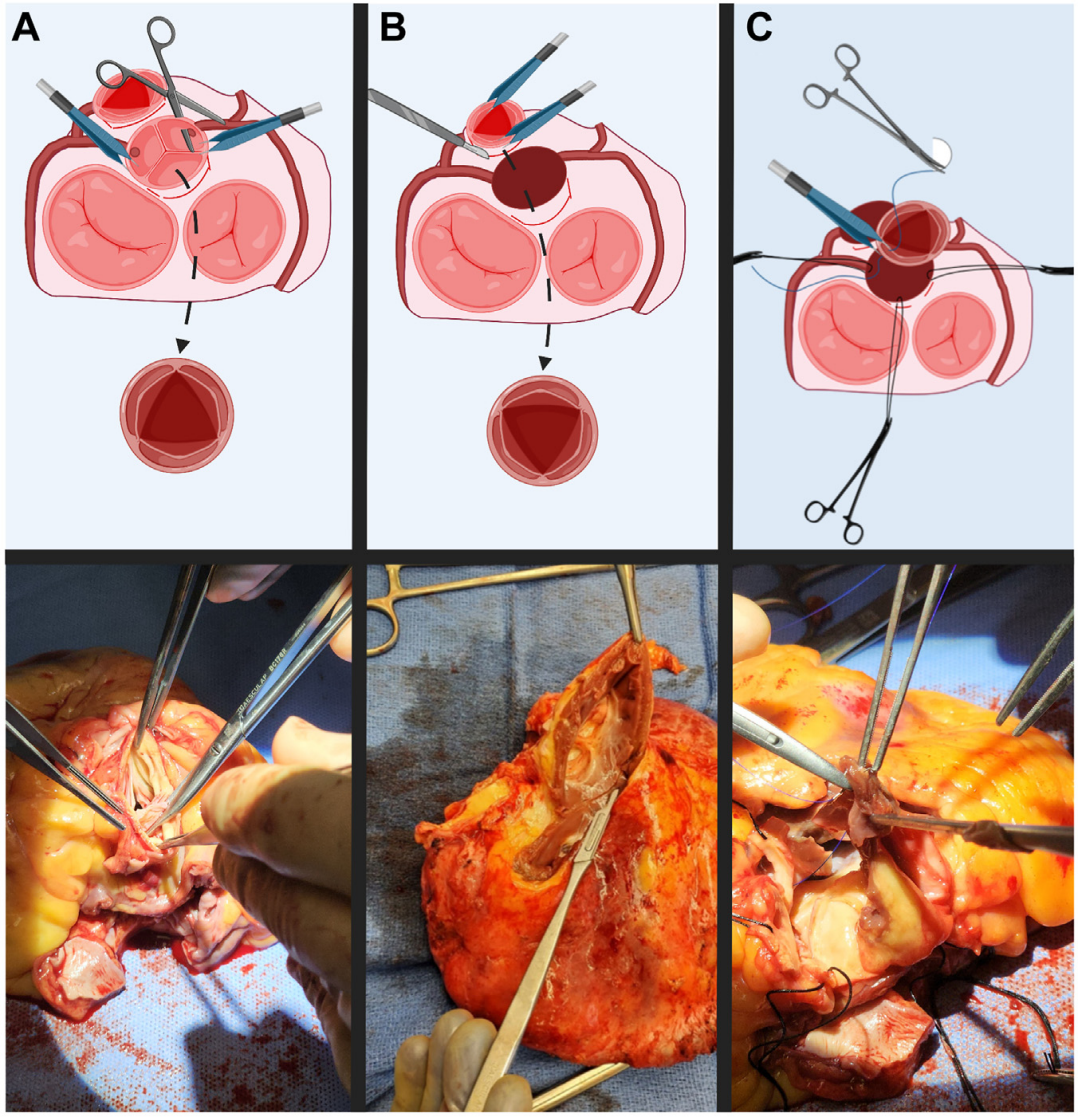
(**A**) Aortic root dissection in preparation for autograft implantation. First, an excision of the aortic valve leaflets is performed. The aortic root is investigated for assessment of annular diameter, commissural symmetry, and height. The coronary buttons are mobilized from the aortic tissue around the ostium. (**B**) Inspection and harvest of the pulmonic valve for autograft harvest. The right ventricular outflow tract is incised, and the pulmonic valve is carefully excised by partially dividing the posterior muscle and taking care to avoid injury to the first septal perforator branch of the left anterior descending artery. The excised pulmonic autograft is carefully inspected, and the infundibular muscle is trimmed to ensure autograft stability. (**C**) Implantation of pulmonic valve autograft. The pulmonic valve is prepared for anastomosis to the neo-aortic root. Silk stay sutures are used for exposure of the aortic root. Autograft implantation is performed using interrupted 4-0 Prolene (Ethicon Inc, Somerville, NJ) or Tri-Cron (Covidien, Mansfield, MA) sutures. The coronary buttons are then reimplanted, with care taken to avoid tension or kinking.

In a native heart, the first step is to incise the PA transversely to inspect the pulmonary valve. If it is adequate, one can proceed with the RP. In the explanted heart, the aorta and PA are already divided. For the sake of simulation, we begin with separation of the PA and the aorta, and then attention is turned toward the left ventricular outflow tract (LVOT). The aortic root and coronary buttons are dissected. The coronary buttons are mobilized to 2-3 mm from the aortic root around the ostium, and the tissue parallel to the aortic wall is dissected down to the level of the right atrium. The aortic valve leaflets are excised, and the root anatomy is examined to investigate annular diameter, commissural symmetry, and height (Fig. 2A). This moment is also opportune to review the anatomy and technical aspects of aortic root enlargement procedures such as the Konno, Nicks-Nunez, and the Manougian. The infundibular ligament between the aorta and PA is divided to complete the mobilization of the aortic root.

We then turn our attention to the excision and preparation of the pulmonic autograft. An area on the RVOT is identified to incise for autograft harvest. A right-angle clamp is placed through the pulmonary valve 6-8 mm below the annulus, and an incision is made at this area (Fig. 2B). The pulmonary valve autograft is then enucleated from the RVOT using Metzenbaum scissors and/or a #15 blade to partially divide the posterior muscle band in the upper part of the infundibular septum, taking care to avoid injury to the first septal perforator branch of the left anterior descending artery (Fig. 2B). The infundibular muscle is trimmed to ensure stability of the autograft; this trimming is particularly important, as the pulmonic valve’s fibrotic annulus is not as well-developed as the native aortic valve.

Once the aortic root is dissected and the pulmonary autograft is prepared, the autograft is anastomosed to the LVOT (Fig. 2C). Now the pulmonic autograft is anastomosed to the LVOT using interrupted or continuous suture with or without use of pericardial strip as a buttress. This step is followed by reimplantation of the left coronary button first, followed by the right coronary button, onto the autograft. The distal anastomoses inherently cannot be simulated. The final step of the RP is replacing the pulmonic valve and the proximal PA with a homograft, which we do not incorporate into our teaching, as this requires costly homografts. However, the principles of homograft preparation and implantation are discussed with participating trainees. Once the simulation is complete, the heart and all dissected tissue are passed off in standard fashion for pathologic analysis directly from the operating room, per institutional policy, without any pathologic concerns. We routinely take high-quality photographs and video recordings of key steps of the RP for future review and education.

## Discussion

Through the use of explanted native hearts during routing OHT to simulate the RP, we aim to familiarize trainees with this complex operation in a risk-free situation. This approach allows trainees to maximize their learning when given the opportunity to participate in an RP in clinical practice. Thus far, we have utilized 6 hearts for RP simulation, with multiple trainees, including general surgical residents and cardiothoracic surgical fellows, who report very high degrees of satisfaction with this educational session.

To our knowledge, this report is the first to describe using fresh hearts explanted at the time of OHT to learn this operation. In addition to the RP, we aim to include additional complex valvular operations to simulate using explanted hearts during OHT. Simulation is instrumental for cardiac surgery training, and high-fidelity models such as 3-dimensional printed or porcine models are instrumental in highlighting key steps of complex operations.^5^ Although animal models are appropriate options for learning and for simulating complex cardiac operations, inherent anatomic differences limit their external generalizability to human patients.^6^ Our approach of using native explanted human hearts for surgical simulation obviates this inherent limitation and allows for realistic simulation with no risk to the patient. Following this proof-of-principle study, we currently are working to recruit trainees to simulate other complex valvular and structural procedures. Our aim is to collect validated pre- and postintervention survey data, to quantitatively assess both the degree of comfort with the procedure and satisfaction with the simulation. Furthermore, we recognize that concomitant simulation of the RP during OHT necessarily requires a larger number of staff in the operating room at one time, and that many transplants occur in the middle of the night. In our practice, the procuring surgeon simulates the RP while on call for procurements in our area. We simulate the RP only when time, patient stability, and resident/fellow availability permit. Important to note is that an inherent limitation of this simulation is the inability to simulate distal anastomoses or implantation of pulmonic homografts. Concomitant simulations in porcine models may be useful for augmenting our simulation to practice these procedures. Furthermore, we acknowledge that we simulate a standard RP as opposed to a supported RP, which consists of the use of a graft prosthesis to support the neo-aortic root. Overall, as technical performance directly influences clinical outcomes after cardiac surgery, our hope is that this high-fidelity approach of teaching the RP will improve patient access to this potentially life-saving operation for younger patients with aortic valve disease requiring surgical replacement.^7^

## Conclusions

The RP is a highly complex operation for aortic valve replacement that uses an autologous pulmonic valve and replacement of the pulmonic valve with a homograft. As a population of younger patients with aortic valvular diseases in need of surgical replacement continues to increase, the training of the next generation of cardiothoracic surgeons in the RP is of paramount importance. Using native hearts explanted at the time of donor cardiectomy during OHT, we demonstrate a novel, high-fidelity model to simulate the RP. Our simulation model incurs no risk to the patient, offers the highest possible resolution with respect to teaching and practicing the operation, and can be reproduced easily at other institutions.
